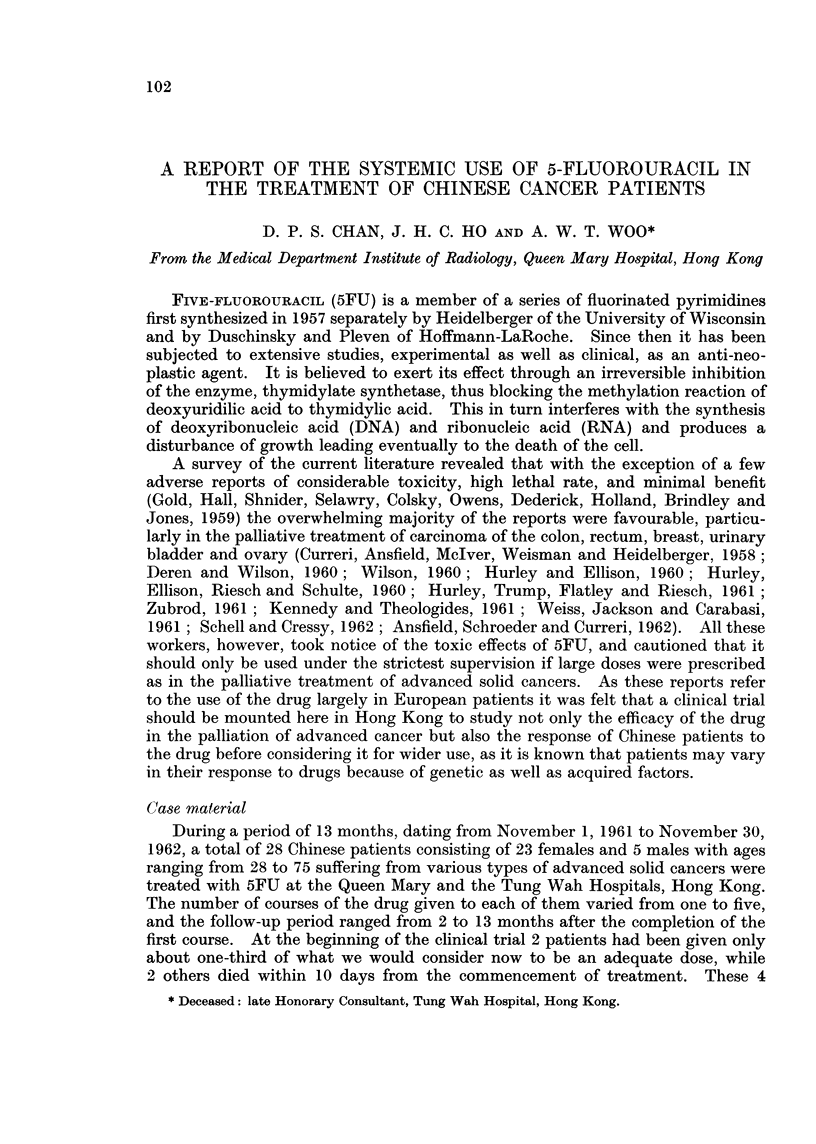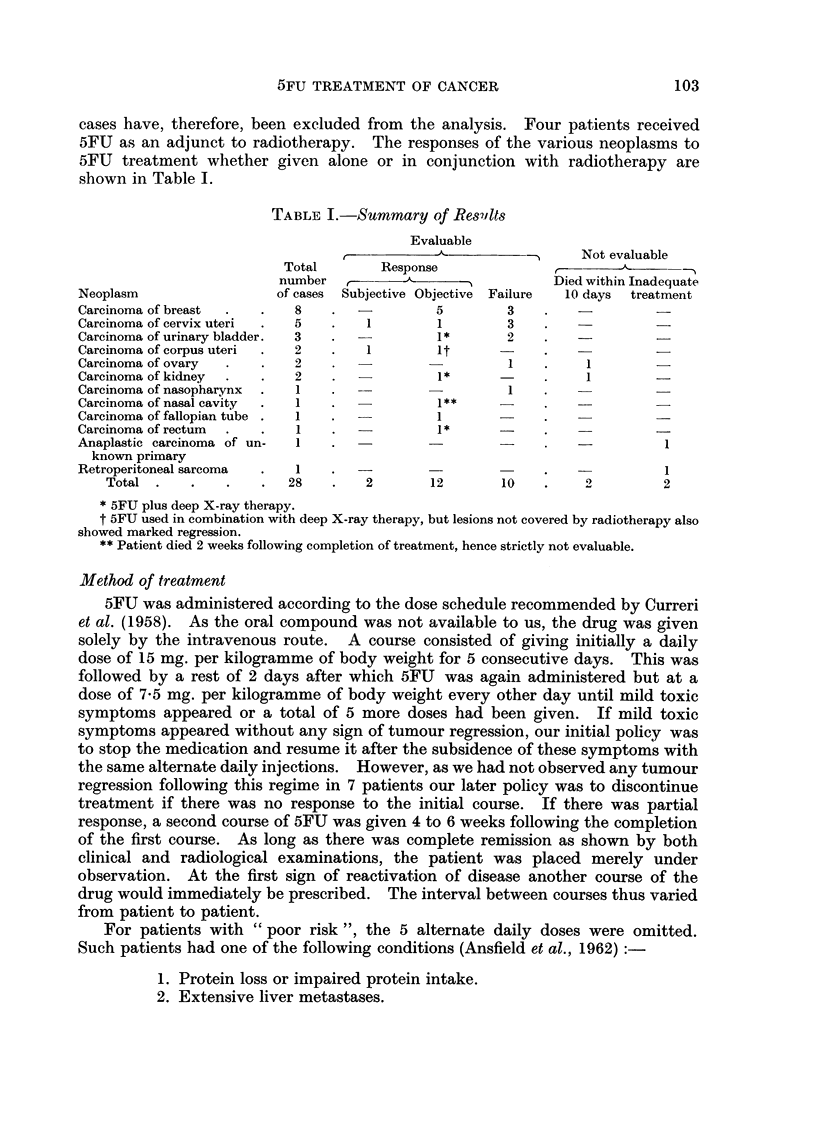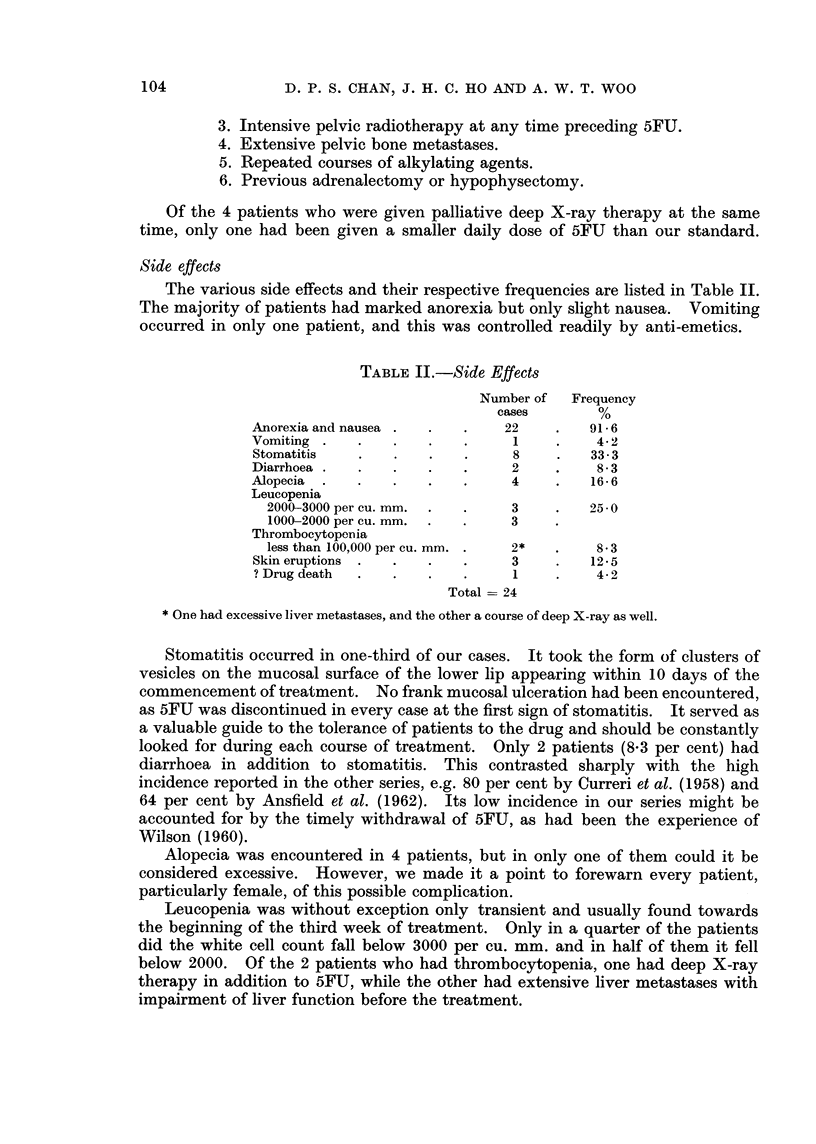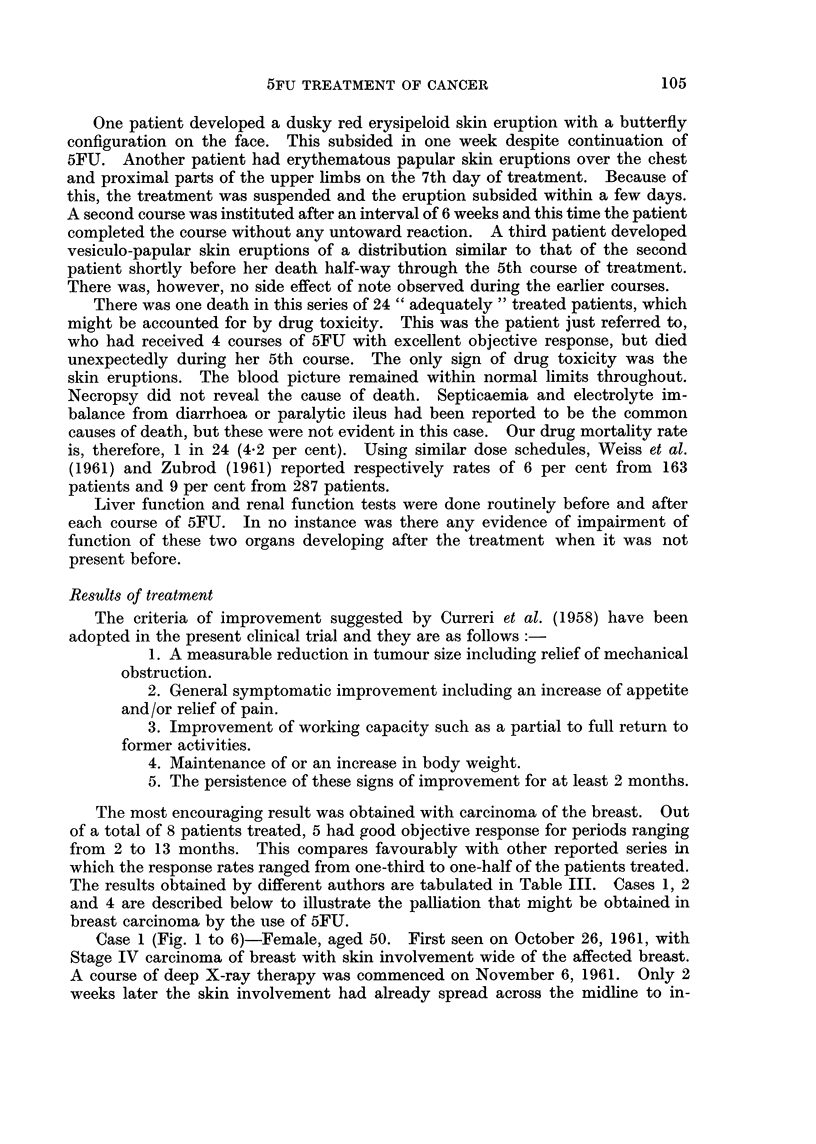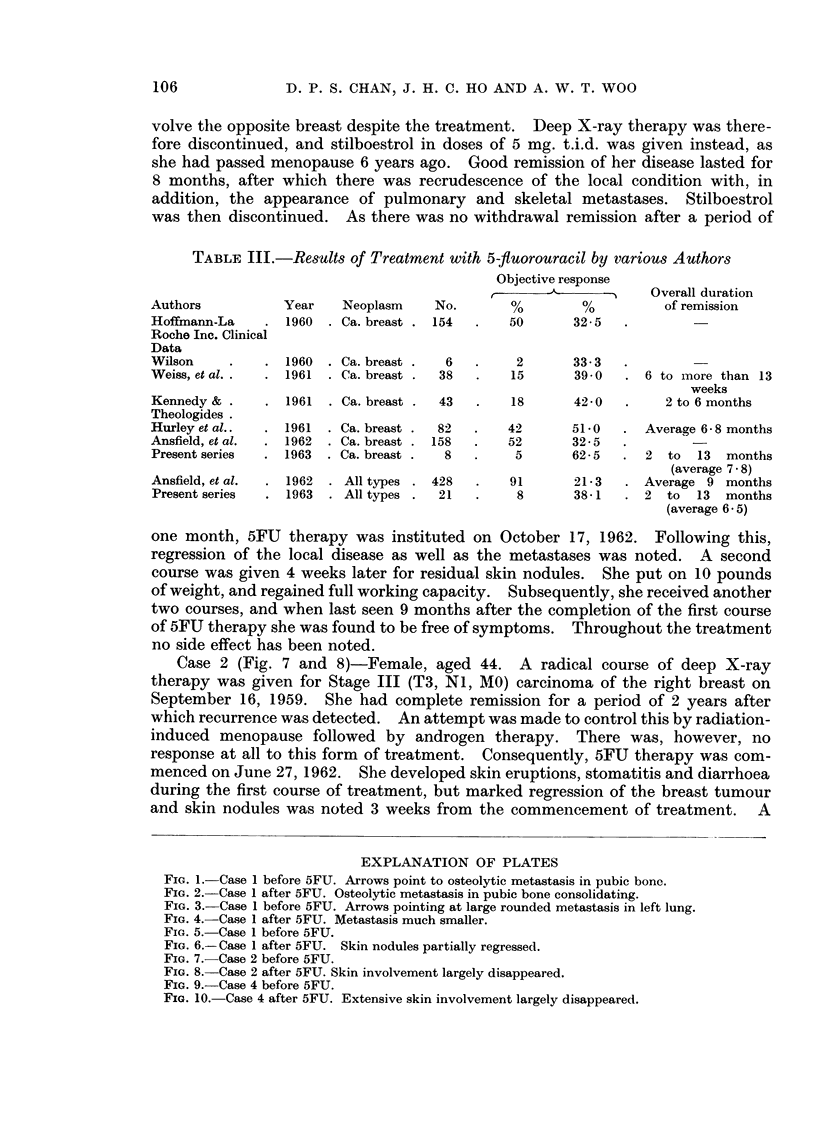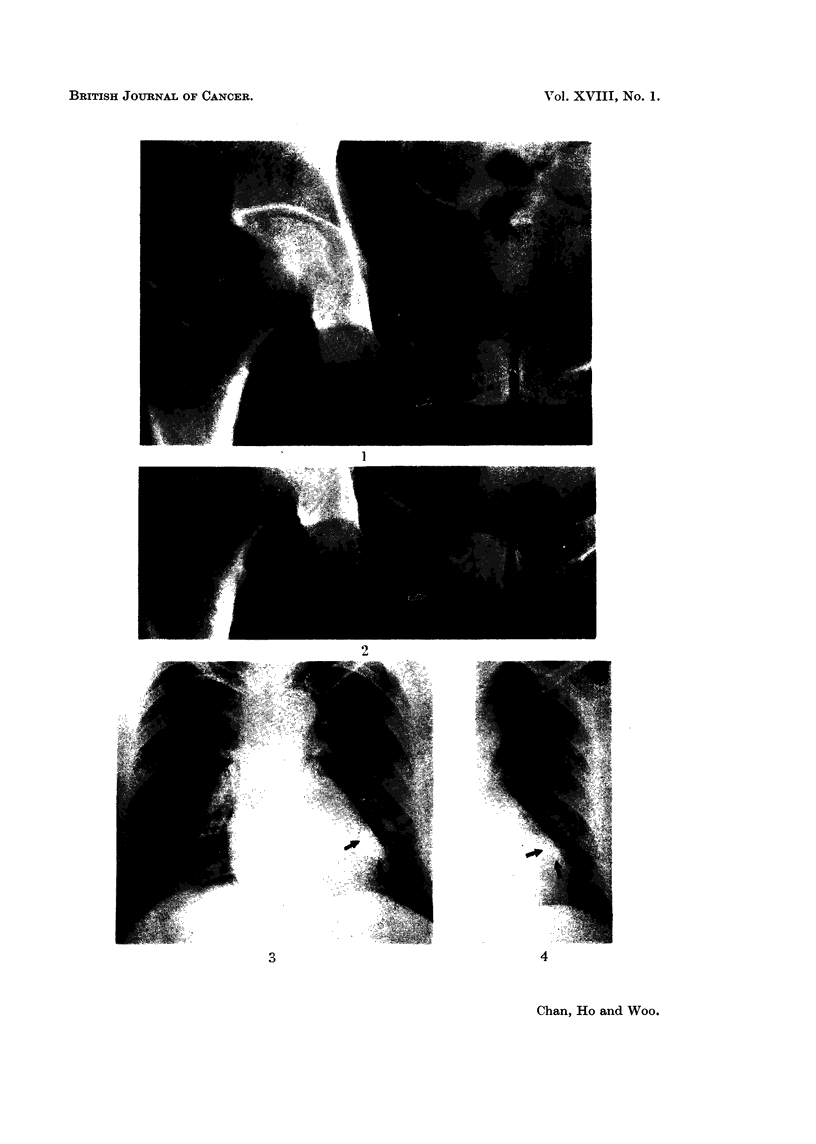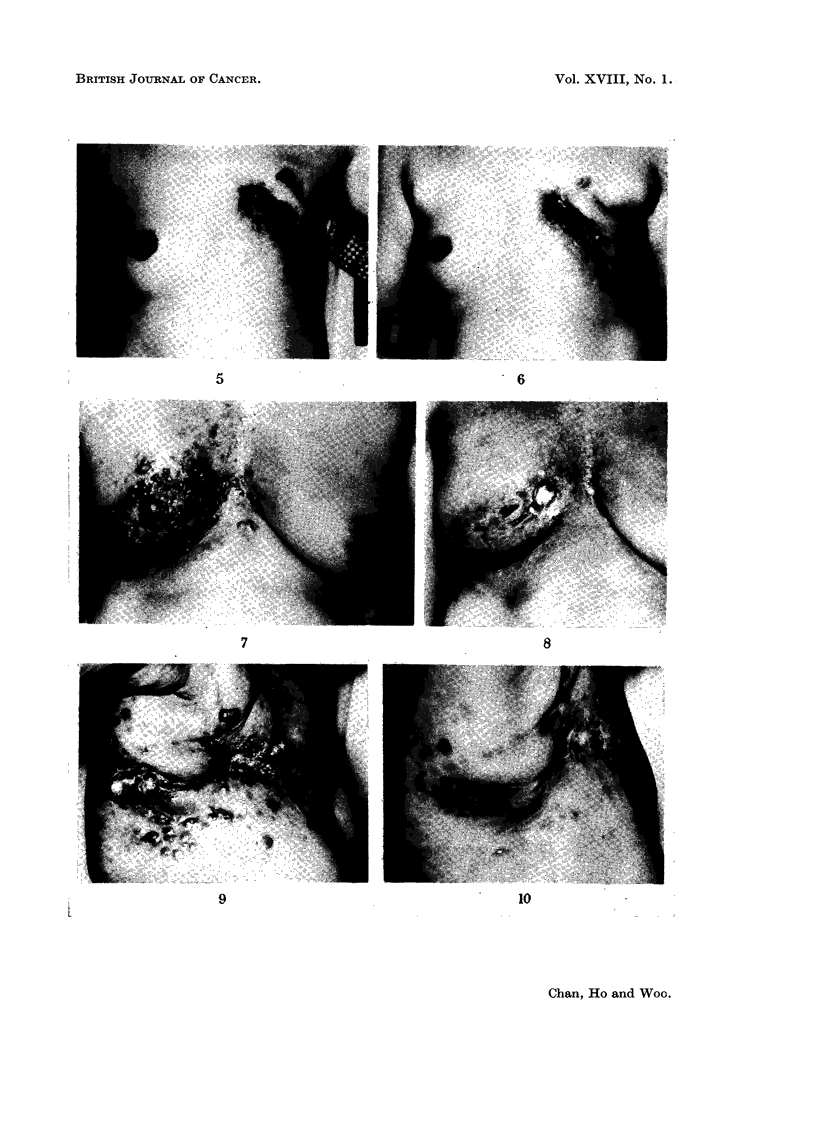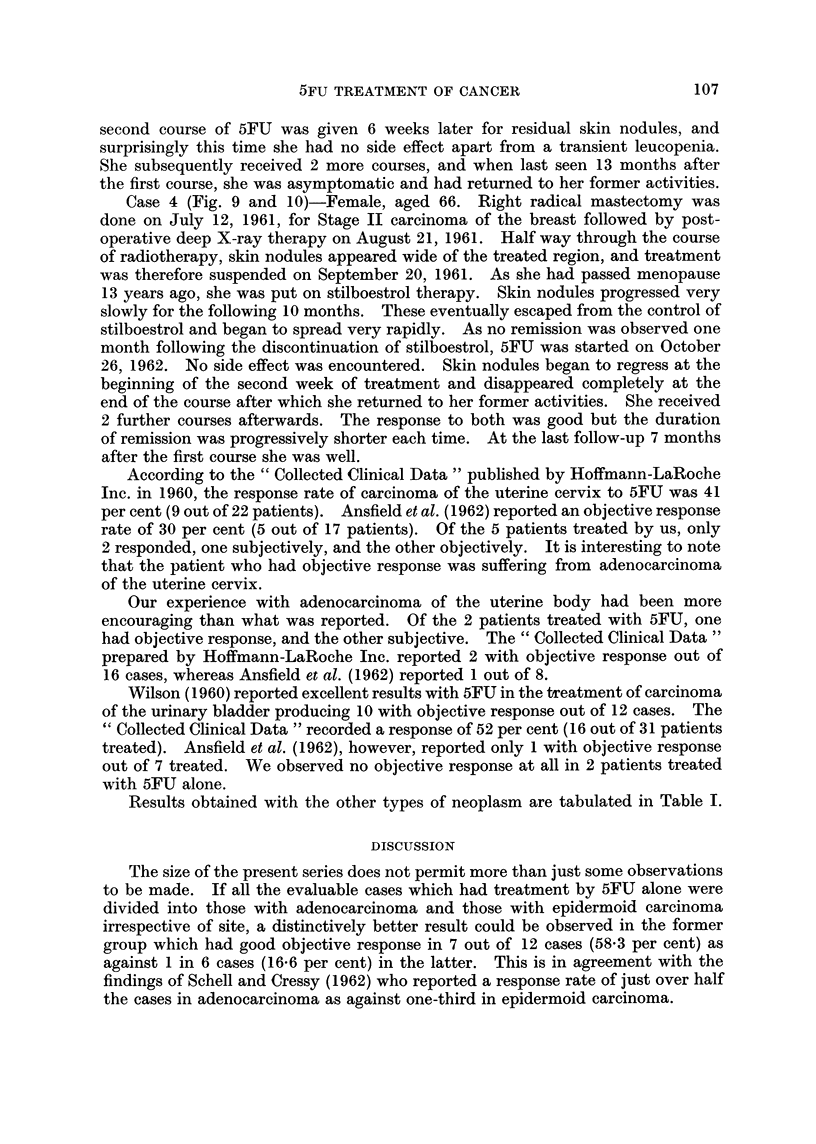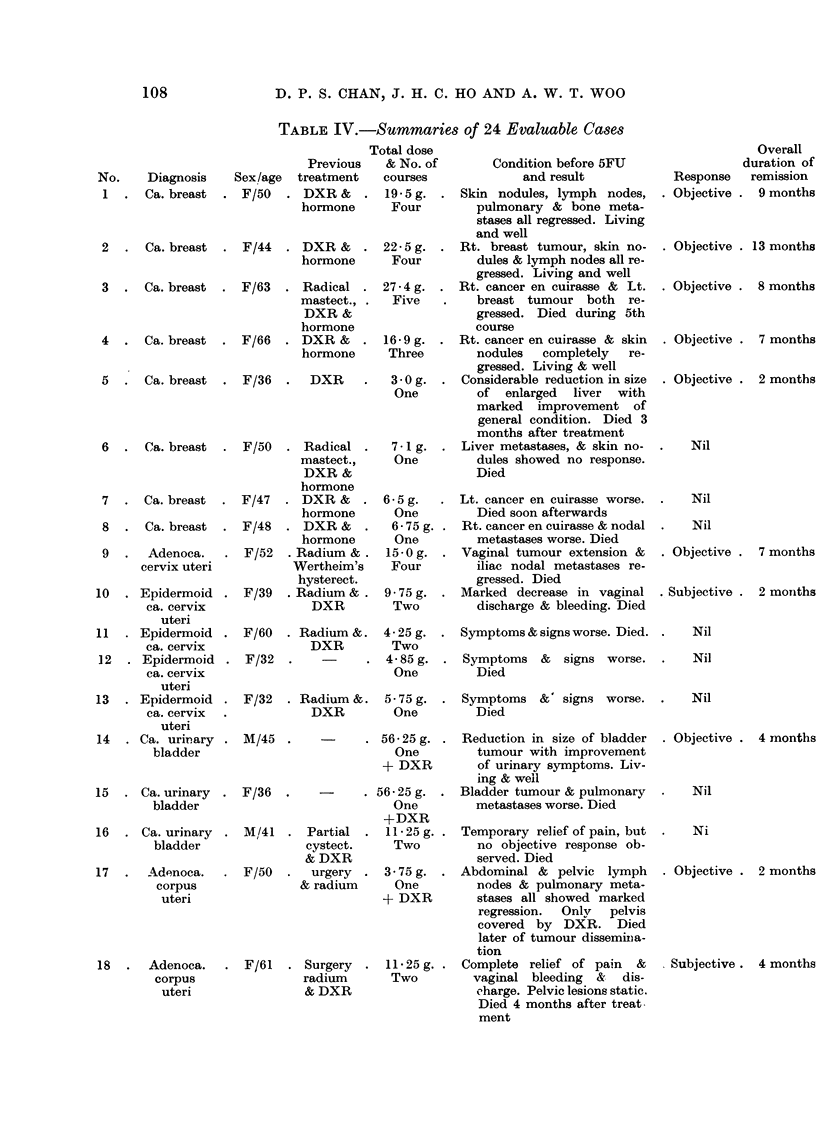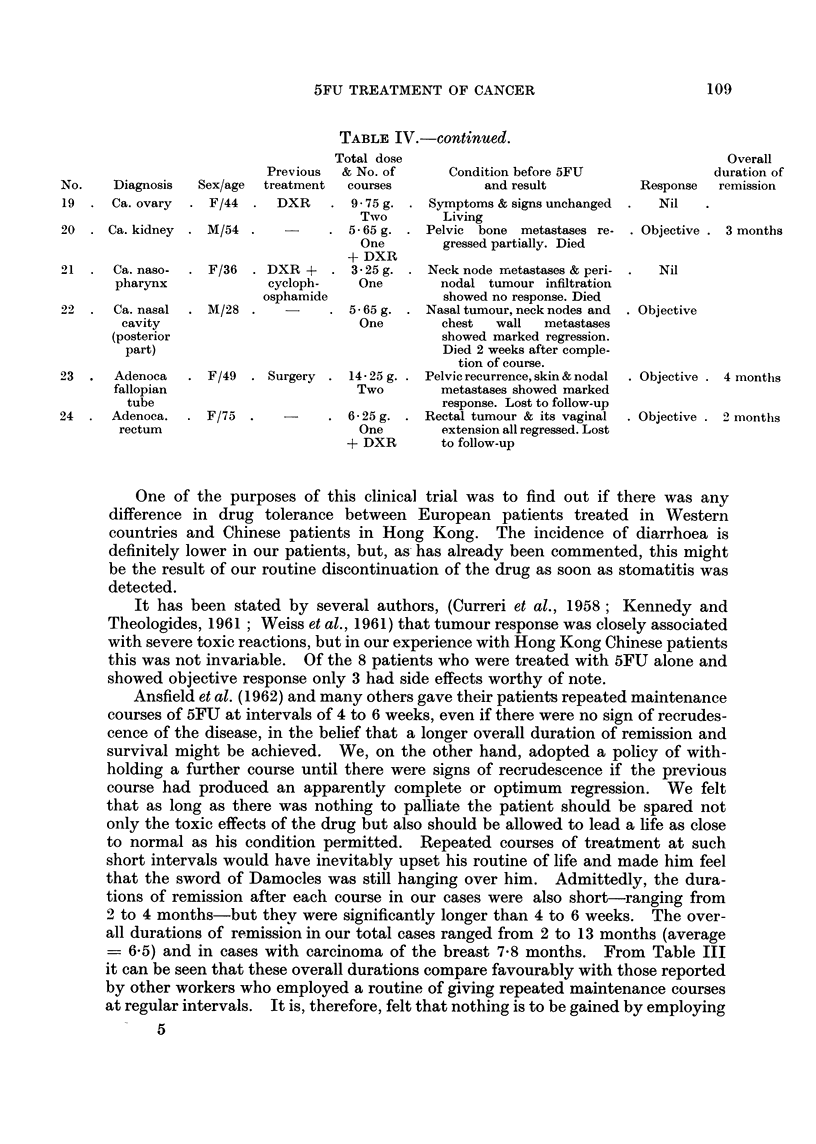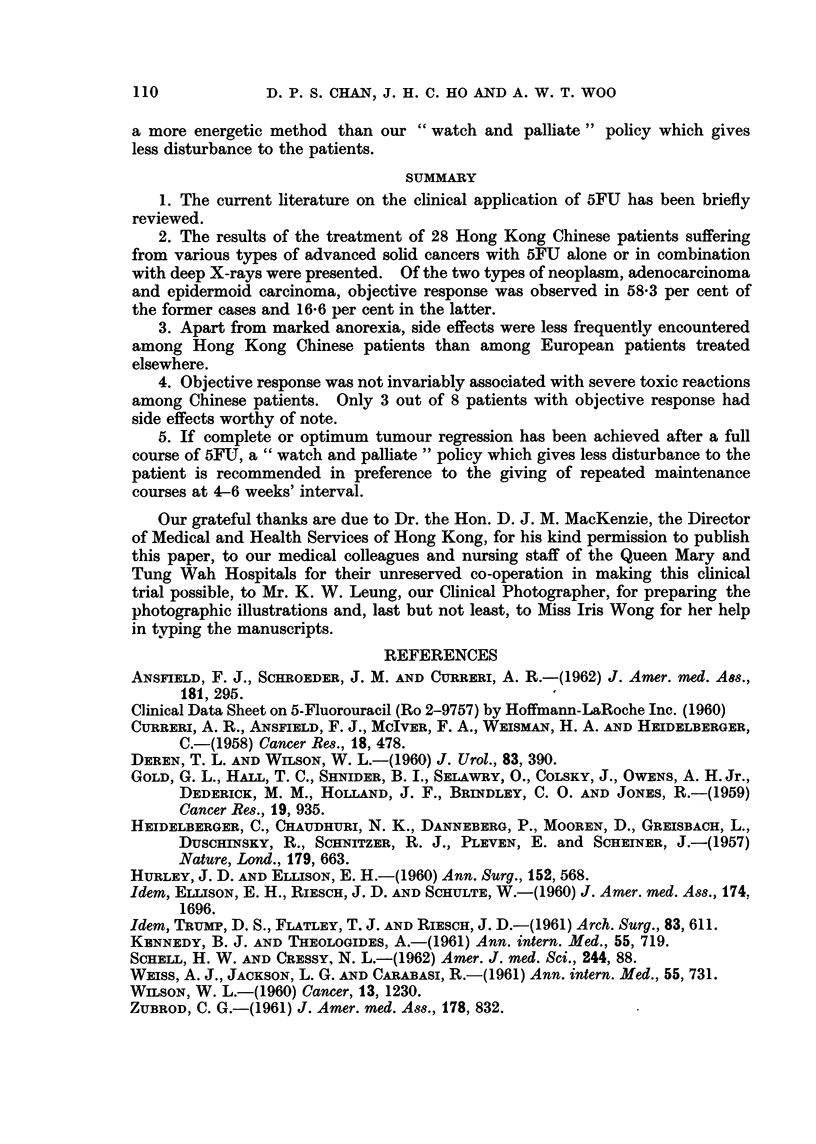# A Report of the Systemic Use of 5-Fluorouracil in the Treatment of Chinese Cancer Patients

**DOI:** 10.1038/bjc.1964.10

**Published:** 1964-03

**Authors:** D. P. S. Chan, J. H. C. Ho, A. W. T. Woo

## Abstract

**Images:**


### A REPORT OF THE SYSTEMIC USE OF 5-FLUOROURACIL IN THE TREATMENT OF CHINESE CANCER PATIENTS

D. P. S. CHAN, J. H. C. HO AND A. W. T. WOO*

*From the Medical Department Institute of Radiology, Queen Mary Hospital, Hong Kong*

FIVE-FLUOROURACIL (5FU) is a member of a series of fluorinated pyrimidines first synthesized in 1957 separately by Heidelberger of the University of Wisconsin and by Duschinsky and Pleven of Hoffmann-LaRoche. Since then it has been subjected to extensive studies, experimental as well as clinical, as an anti-neoplastic agent. It is believed to exert its effect through an irreversible inhibition of the enzyme, thymidylate synthetase, thus blocking the methylation reaction of deoxyuridilic acid to thymidylic acid. This in turn interferes with the synthesis of deoxyribonucleic acid (DNA) and ribonucleic acid (RNA) and produces a disturbance of growth leading eventually to the death of the cell.

A survey of the current literature revealed that with the exception of a few adverse reports of considerable toxicity, high lethal rate, and minimal benefit (Gold, Hall, Shnider, Selawry, Colsky, Owens, Dederick, Holland, Brindley and Jones, 1959) the overwhelming majority of the reports were favourable, particularly in the palliative treatment of carcinoma of the colon, rectum, breast, urinary bladder and ovary (Curreri, Ansfield, McIver, Weisman and Heidelberger, 1958 ; Deren and Wilson, 1960 ; Wilson, 1960 ; Hurley and Ellison, 1960 ; Hurley, Ellison, Riesch and Schulte, 1960 ; Hurley, Trump, Flatley and Riesch, 1961 ; Zubrod, 1961 ; Kennedy and Theologides, 1961 ; Weiss, Jackson and Carabasi, 1961 ; Schell and Cressy, 1962 ; Ansfield, Schroeder and Curreri, 1962). All these workers, however, took notice of the toxic effects of 5FU, and cautioned that it should only be used under the strictest supervision if large doses were prescribed as in the palliative treatment of advanced solid cancers. As these reports refer to the use of the drug largely in European patients it was felt that a clinical trial should be mounted here in Hong Kong to study not only the efficacy of the drug in the palliation of advanced cancer but also the response of Chinese patients to the drug before considering it for wider use, as it is known that patients may vary in their response to drugs because of genetic as well as acquired factors.

### *Case material*

During a period of 13 months, dating from November 1, 1961 to November 30, 1962, a total of 28 Chinese patients consisting of 23 females and 5 males with ages ranging from 28 to 75 suffering from various types of advanced solid cancers were treated with 5FU at the Queen Mary and the Tung Wah Hospitals, Hong Kong. The number of courses of the drug given to each of them varied from one to five, and the follow-up period ranged from 2 to 13 months after the completion of the first course. At the beginning of the clinical trial 2 patients had been given only about one-third of what we would consider now to be an adequate dose, while 2 others died within 10 days from the commencement of treatment. These 4

* Deceased: late Honorary Consultant, Tung Wah Hospital, Hong Kong.

cases have, therefore, been excluded from the analysis. Four patients received 5FU as an adjunct to radiotherapy. The responses of the various neoplasms to 5FU treatment whether given alone or in conjunction with radiotherapy are shown in Table I.

TABLE I.—*Summary of Results*

| Neoplasm | Total number of cases | Evaluable: Response | | | Not evaluable | |
|---|---|---|---|---|---|---|
| | | Subjective | Objective | Failure | Died within 10 days | Inadequate treatment |
| Carcinoma of breast | 8 | — | 5 | 3 | — | — |
| Carcinoma of cervix uteri | 5 | 1 | 1 | 3 | — | — |
| Carcinoma of urinary bladder | 3 | — | 1* | 2 | — | — |
| Carcinoma of corpus uteri | 2 | 1 | 1† | — | — | — |
| Carcinoma of ovary | 2 | — | — | 1 | 1 | — |
| Carcinoma of kidney | 2 | — | 1* | — | 1 | — |
| Carcinoma of nasopharynx | 1 | — | — | 1 | — | — |
| Carcinoma of nasal cavity | 1 | — | 1** | — | — | — |
| Carcinoma of fallopian tube | 1 | — | 1 | — | — | — |
| Carcinoma of rectum | 1 | — | 1* | — | — | — |
| Anaplastic carcinoma of unknown primary | 1 | — | — | — | — | 1 |
| Retroperitoneal sarcoma | 1 | — | — | — | — | 1 |
| Total | 28 | 2 | 12 | 10 | 2 | 2 |

* 5FU plus deep X-ray therapy.
† 5FU used in combination with deep X-ray therapy, but lesions not covered by radiotherapy also showed marked regression.
** Patient died 2 weeks following completion of treatment, hence strictly not evaluable.

*Method of treatment*

5FU was administered according to the dose schedule recommended by Curreri *et al.* (1958). As the oral compound was not available to us, the drug was given solely by the intravenous route. A course consisted of giving initially a daily dose of 15 mg. per kilogramme of body weight for 5 consecutive days. This was followed by a rest of 2 days after which 5FU was again administered but at a dose of 7·5 mg. per kilogramme of body weight every other day until mild toxic symptoms appeared or a total of 5 more doses had been given. If mild toxic symptoms appeared without any sign of tumour regression, our initial policy was to stop the medication and resume it after the subsidence of these symptoms with the same alternate daily injections. However, as we had not observed any tumour regression following this regime in 7 patients our later policy was to discontinue treatment if there was no response to the initial course. If there was partial response, a second course of 5FU was given 4 to 6 weeks following the completion of the first course. As long as there was complete remission as shown by both clinical and radiological examinations, the patient was placed merely under observation. At the first sign of reactivation of disease another course of the drug would immediately be prescribed. The interval between courses thus varied from patient to patient.

For patients with " poor risk ", the 5 alternate daily doses were omitted. Such patients had one of the following conditions (Ansfield *et al.*, 1962) :—

1. Protein loss or impaired protein intake.
2. Extensive liver metastases.

3. Intensive pelvic radiotherapy at any time preceding 5FU.
4. Extensive pelvic bone metastases.
5. Repeated courses of alkylating agents.
6. Previous adrenalectomy or hypophysectomy.

Of the 4 patients who were given palliative deep X-ray therapy at the same time, only one had been given a smaller daily dose of 5FU than our standard.

*Side effects*

The various side effects and their respective frequencies are listed in Table II. The majority of patients had marked anorexia but only slight nausea. Vomiting occurred in only one patient, and this was controlled readily by anti-emetics.

TABLE II.—*Side Effects*

| | Number of cases | Frequency % |
|---|---|---|
| Anorexia and nausea | 22 | 91·6 |
| Vomiting | 1 | 4·2 |
| Stomatitis | 8 | 33·3 |
| Diarrhoea | 2 | 8·3 |
| Alopecia | 4 | 16·6 |
| Leucopenia | | |
| 2000–3000 per cu. mm. | 3 | 25·0 |
| 1000–2000 per cu. mm. | 3 | |
| Thrombocytopenia | | |
| less than 100,000 per cu. mm. | 2* | 8·3 |
| Skin eruptions | 3 | 12·5 |
| ? Drug death | 1 | 4·2 |
| | Total = 24 | |

* One had excessive liver metastases, and the other a course of deep X-ray as well.

Stomatitis occurred in one-third of our cases. It took the form of clusters of vesicles on the mucosal surface of the lower lip appearing within 10 days of the commencement of treatment. No frank mucosal ulceration had been encountered, as 5FU was discontinued in every case at the first sign of stomatitis. It served as a valuable guide to the tolerance of patients to the drug and should be constantly looked for during each course of treatment. Only 2 patients (8·3 per cent) had diarrhoea in addition to stomatitis. This contrasted sharply with the high incidence reported in the other series, e.g. 80 per cent by Curreri *et al.* (1958) and 64 per cent by Ansfield *et al.* (1962). Its low incidence in our series might be accounted for by the timely withdrawal of 5FU, as had been the experience of Wilson (1960).

Alopecia was encountered in 4 patients, but in only one of them could it be considered excessive. However, we made it a point to forewarn every patient, particularly female, of this possible complication.

Leucopenia was without exception only transient and usually found towards the beginning of the third week of treatment. Only in a quarter of the patients did the white cell count fall below 3000 per cu. mm. and in half of them it fell below 2000. Of the 2 patients who had thrombocytopenia, one had deep X-ray therapy in addition to 5FU, while the other had extensive liver metastases with impairment of liver function before the treatment.

One patient developed a dusky red erysipeloid skin eruption with a butterfly configuration on the face. This subsided in one week despite continuation of 5FU. Another patient had erythematous papular skin eruptions over the chest and proximal parts of the upper limbs on the 7th day of treatment. Because of this, the treatment was suspended and the eruption subsided within a few days. A second course was instituted after an interval of 6 weeks and this time the patient completed the course without any untoward reaction. A third patient developed vesiculo-papular skin eruptions of a distribution similar to that of the second patient shortly before her death half-way through the 5th course of treatment. There was, however, no side effect of note observed during the earlier courses.

There was one death in this series of 24 " adequately " treated patients, which might be accounted for by drug toxicity. This was the patient just referred to, who had received 4 courses of 5FU with excellent objective response, but died unexpectedly during her 5th course. The only sign of drug toxicity was the skin eruptions. The blood picture remained within normal limits throughout. Necropsy did not reveal the cause of death. Septicaemia and electrolyte imbalance from diarrhoea or paralytic ileus had been reported to be the common causes of death, but these were not evident in this case. Our drug mortality rate is, therefore, 1 in 24 (4·2 per cent). Using similar dose schedules, Weiss *et al.* (1961) and Zubrod (1961) reported respectively rates of 6 per cent from 163 patients and 9 per cent from 287 patients.

Liver function and renal function tests were done routinely before and after each course of 5FU. In no instance was there any evidence of impairment of function of these two organs developing after the treatment when it was not present before.

*Results of treatment*

The criteria of improvement suggested by Curreri *et al.* (1958) have been adopted in the present clinical trial and they are as follows :—

1. A measurable reduction in tumour size including relief of mechanical obstruction.
2. General symptomatic improvement including an increase of appetite and/or relief of pain.
3. Improvement of working capacity such as a partial to full return to former activities.
4. Maintenance of or an increase in body weight.
5. The persistence of these signs of improvement for at least 2 months.

The most encouraging result was obtained with carcinoma of the breast. Out of a total of 8 patients treated, 5 had good objective response for periods ranging from 2 to 13 months. This compares favourably with other reported series in which the response rates ranged from one-third to one-half of the patients treated. The results obtained by different authors are tabulated in Table III. Cases 1, 2 and 4 are described below to illustrate the palliation that might be obtained in breast carcinoma by the use of 5FU.

Case 1 (Fig. 1 to 6)—Female, aged 50. First seen on October 26, 1961, with Stage IV carcinoma of breast with skin involvement wide of the affected breast. A course of deep X-ray therapy was commenced on November 6, 1961. Only 2 weeks later the skin involvement had already spread across the midline to involve the opposite breast despite the treatment. Deep X-ray therapy was therefore discontinued, and stilboestrol in doses of 5 mg. t.i.d. was given instead, as she had passed menopause 6 years ago. Good remission of her disease lasted for 8 months, after which there was recrudescence of the local condition with, in addition, the appearance of pulmonary and skeletal metastases. Stilboestrol was then discontinued. As there was no withdrawal remission after a period of

TABLE III.—*Results of Treatment with 5-fluorouracil by various Authors*

| Authors | Year | Neoplasm | No. | Objective response % | Objective response % | Overall duration of remission |
|---|---|---|---|---|---|---|
| Hoffmann-La Roche Inc. Clinical Data | 1960 | Ca. breast | 154 | 50 | 32·5 | — |
| Wilson | 1960 | Ca. breast | 6 | 2 | 33·3 | — |
| Weiss, *et al.* | 1961 | Ca. breast | 38 | 15 | 39·0 | 6 to more than 13 weeks |
| Kennedy & Theologides | 1961 | Ca. breast | 43 | 18 | 42·0 | 2 to 6 months |
| Hurley *et al.* | 1961 | Ca. breast | 82 | 42 | 51·0 | Average 6·8 months |
| Ansfield, *et al.* | 1962 | Ca. breast | 158 | 52 | 32·5 | — |
| Present series | 1963 | Ca. breast | 8 | 5 | 62·5 | 2 to 13 months (average 7·8) |
| Ansfield, *et al.* | 1962 | All types | 428 | 91 | 21·3 | Average 9 months |
| Present series | 1963 | All types | 21 | 8 | 38·1 | 2 to 13 months (average 6·5) |

one month, 5FU therapy was instituted on October 17, 1962. Following this, regression of the local disease as well as the metastases was noted. A second course was given 4 weeks later for residual skin nodules. She put on 10 pounds of weight, and regained full working capacity. Subsequently, she received another two courses, and when last seen 9 months after the completion of the first course of 5FU therapy she was found to be free of symptoms. Throughout the treatment no side effect has been noted.

Case 2 (Fig. 7 and 8)—Female, aged 44. A radical course of deep X-ray therapy was given for Stage III (T3, N1, M0) carcinoma of the right breast on September 16, 1959. She had complete remission for a period of 2 years after which recurrence was detected. An attempt was made to control this by radiation-induced menopause followed by androgen therapy. There was, however, no response at all to this form of treatment. Consequently, 5FU therapy was commenced on June 27, 1962. She developed skin eruptions, stomatitis and diarrhoea during the first course of treatment, but marked regression of the breast tumour and skin nodules was noted 3 weeks from the commencement of treatment. A

EXPLANATION OF PLATES

FIG. 1.—Case 1 before 5FU. Arrows point to osteolytic metastasis in pubic bone.
FIG. 2.—Case 1 after 5FU. Osteolytic metastasis in pubic bone consolidating.
FIG. 3.—Case 1 before 5FU. Arrows pointing at large rounded metastasis in left lung.
FIG. 4.—Case 1 after 5FU. Metastasis much smaller.
FIG. 5.—Case 1 before 5FU.
FIG. 6.—Case 1 after 5FU. Skin nodules partially regressed.
FIG. 7.—Case 2 before 5FU.
FIG. 8.—Case 2 after 5FU. Skin involvement largely disappeared.
FIG. 9.—Case 4 before 5FU.
FIG. 10.—Case 4 after 5FU. Extensive skin involvement largely disappeared.

1

2

3

4

Chan, Ho and Woo.

5

6

7

8

9

10

second course of 5FU was given 6 weeks later for residual skin nodules, and surprisingly this time she had no side effect apart from a transient leucopenia. She subsequently received 2 more courses, and when last seen 13 months after the first course, she was asymptomatic and had returned to her former activities.

Case 4 (Fig. 9 and 10)—Female, aged 66. Right radical mastectomy was done on July 12, 1961, for Stage II carcinoma of the breast followed by post-operative deep X-ray therapy on August 21, 1961. Half way through the course of radiotherapy, skin nodules appeared wide of the treated region, and treatment was therefore suspended on September 20, 1961. As she had passed menopause 13 years ago, she was put on stilboestrol therapy. Skin nodules progressed very slowly for the following 10 months. These eventually escaped from the control of stilboestrol and began to spread very rapidly. As no remission was observed one month following the discontinuation of stilboestrol, 5FU was started on October 26, 1962. No side effect was encountered. Skin nodules began to regress at the beginning of the second week of treatment and disappeared completely at the end of the course after which she returned to her former activities. She received 2 further courses afterwards. The response to both was good but the duration of remission was progressively shorter each time. At the last follow-up 7 months after the first course she was well.

According to the " Collected Clinical Data " published by Hoffmann-LaRoche Inc. in 1960, the response rate of carcinoma of the uterine cervix to 5FU was 41 per cent (9 out of 22 patients). Ansfield *et al.* (1962) reported an objective response rate of 30 per cent (5 out of 17 patients). Of the 5 patients treated by us, only 2 responded, one subjectively, and the other objectively. It is interesting to note that the patient who had objective response was suffering from adenocarcinoma of the uterine cervix.

Our experience with adenocarcinoma of the uterine body had been more encouraging than what was reported. Of the 2 patients treated with 5FU, one had objective response, and the other subjective. The " Collected Clinical Data " prepared by Hoffmann-LaRoche Inc. reported 2 with objective response out of 16 cases, whereas Ansfield *et al.* (1962) reported 1 out of 8.

Wilson (1960) reported excellent results with 5FU in the treatment of carcinoma of the urinary bladder producing 10 with objective response out of 12 cases. The " Collected Clinical Data " recorded a response of 52 per cent (16 out of 31 patients treated). Ansfield *et al.* (1962), however, reported only 1 with objective response out of 7 treated. We observed no objective response at all in 2 patients treated with 5FU alone.

Results obtained with the other types of neoplasm are tabulated in Table I.

### DISCUSSION

The size of the present series does not permit more than just some observations to be made. If all the evaluable cases which had treatment by 5FU alone were divided into those with adenocarcinoma and those with epidermoid carcinoma irrespective of site, a distinctively better result could be observed in the former group which had good objective response in 7 out of 12 cases (58·3 per cent) as against 1 in 6 cases (16·6 per cent) in the latter. This is in agreement with the findings of Schell and Cressy (1962) who reported a response rate of just over half the cases in adenocarcinoma as against one-third in epidermoid carcinoma.

TABLE IV.—*Summaries of 24 Evaluable Cases*

| No. | Diagnosis | Sex/age | Previous treatment | Total dose & No. of courses | Condition before 5FU and result | Response | Overall duration of remission |
| --- | --- | --- | --- | --- | --- | --- | --- |
| 1 | Ca. breast | F/50 | DXR & hormone | 19·5 g. Four | Skin nodules, lymph nodes, pulmonary & bone metastases all regressed. Living and well | Objective | 9 months |
| 2 | Ca. breast | F/44 | DXR & hormone | 22·5 g. Four | Rt. breast tumour, skin nodules & lymph nodes all regressed. Living and well | Objective | 13 months |
| 3 | Ca. breast | F/63 | Radical mastect., DXR & hormone | 27·4 g. Five | Rt. cancer en cuirasse & Lt. breast tumour both regressed. Died during 5th course | Objective | 8 months |
| 4 | Ca. breast | F/66 | DXR & hormone | 16·9 g. Three | Rt. cancer en cuirasse & skin nodules completely regressed. Living & well | Objective | 7 months |
| 5 | Ca. breast | F/36 | DXR | 3·0 g. One | Considerable reduction in size of enlarged liver with marked improvement of general condition. Died 3 months after treatment | Objective | 2 months |
| 6 | Ca. breast | F/50 | Radical mastect., DXR & hormone | 7·1 g. One | Liver metastases, & skin nodules showed no response. Died | Nil | |
| 7 | Ca. breast | F/47 | DXR & hormone | 6·5 g. One | Lt. cancer en cuirasse worse. Died soon afterwards | Nil | |
| 8 | Ca. breast | F/48 | DXR & hormone | 6·75 g. One | Rt. cancer en cuirasse & nodal metastases worse. Died | Nil | |
| 9 | Adenoca. cervix uteri | F/52 | Radium & Wertheim's hysterect. | 15·0 g. Four | Vaginal tumour extension & iliac nodal metastases regressed. Died | Objective | 7 months |
| 10 | Epidermoid ca. cervix uteri | F/39 | Radium & DXR | 9·75 g. Two | Marked decrease in vaginal discharge & bleeding. Died | Subjective | 2 months |
| 11 | Epidermoid ca. cervix | F/60 | Radium & DXR | 4·25 g. Two | Symptoms & signs worse. Died. | Nil | |
| 12 | Epidermoid ca. cervix uteri | F/32 | — | 4·85 g. One | Symptoms & signs worse. Died | Nil | |
| 13 | Epidermoid ca. cervix uteri | F/32 | Radium & DXR | 5·75 g. One | Symptoms & signs worse. Died | Nil | |
| 14 | Ca. urinary bladder | M/45 | — | 56·25 g. One + DXR | Reduction in size of bladder tumour with improvement of urinary symptoms. Living & well | Objective | 4 months |
| 15 | Ca. urinary bladder | F/36 | — | 56·25 g. One +DXR | Bladder tumour & pulmonary metastases worse. Died | Nil | |
| 16 | Ca. urinary bladder | M/41 | Partial cystect. & DXR | 11·25 g. Two | Temporary relief of pain, but no objective response observed. Died | Ni | |
| 17 | Adenoca. corpus uteri | F/50 | urgery & radium | 3·75 g. One + DXR | Abdominal & pelvic lymph nodes & pulmonary metastases all showed marked regression. Only pelvis covered by DXR. Died later of tumour dissemination | Objective | 2 months |
| 18 | Adenoca. corpus uteri | F/61 | Surgery radium & DXR | 11·25 g. Two | Complete relief of pain & vaginal bleeding & discharge. Pelvic lesions static. Died 4 months after treatment | Subjective | 4 months |

TABLE IV.—*continued.*

| No. | Diagnosis | Sex/age | Previous treatment | Total dose & No. of courses | Condition before 5FU and result | Response | Overall duration of remission |
|---|---|---|---|---|---|---|---|
| 19 | Ca. ovary | F/44 | DXR | 9·75 g. Two | Symptoms & signs unchanged Living | Nil | |
| 20 | Ca. kidney | M/54 | — | 5·65 g. One + DXR | Pelvic bone metastases regressed partially. Died | Objective | 3 months |
| 21 | Ca. nasopharynx | F/36 | DXR + cyclophosphamide | 3·25 g. One | Neck node metastases & perinodal tumour infiltration showed no response. Died | Nil | |
| 22 | Ca. nasal cavity (posterior part) | M/28 | — | 5·65 g. One | Nasal tumour, neck nodes and chest wall metastases showed marked regression. Died 2 weeks after completion of course. | Objective | |
| 23 | Adenoca fallopian tube | F/49 | Surgery | 14·25 g. Two | Pelvic recurrence, skin & nodal metastases showed marked response. Lost to follow-up | Objective | 4 months |
| 24 | Adenoca. rectum | F/75 | — | 6·25 g. One + DXR | Rectal tumour & its vaginal extension all regressed. Lost to follow-up | Objective | 2 months |

One of the purposes of this clinical trial was to find out if there was any difference in drug tolerance between European patients treated in Western countries and Chinese patients in Hong Kong. The incidence of diarrhoea is definitely lower in our patients, but, as has already been commented, this might be the result of our routine discontinuation of the drug as soon as stomatitis was detected.

It has been stated by several authors, (Curreri *et al.*, 1958 ; Kennedy and Theologides, 1961 ; Weiss *et al.*, 1961) that tumour response was closely associated with severe toxic reactions, but in our experience with Hong Kong Chinese patients this was not invariable. Of the 8 patients who were treated with 5FU alone and showed objective response only 3 had side effects worthy of note.

Ansfield *et al.* (1962) and many others gave their patients repeated maintenance courses of 5FU at intervals of 4 to 6 weeks, even if there were no sign of recrudescence of the disease, in the belief that a longer overall duration of remission and survival might be achieved. We, on the other hand, adopted a policy of withholding a further course until there were signs of recrudescence if the previous course had produced an apparently complete or optimum regression. We felt that as long as there was nothing to palliate the patient should be spared not only the toxic effects of the drug but also should be allowed to lead a life as close to normal as his condition permitted. Repeated courses of treatment at such short intervals would have inevitably upset his routine of life and made him feel that the sword of Damocles was still hanging over him. Admittedly, the durations of remission after each course in our cases were also short—ranging from 2 to 4 months—but they were significantly longer than 4 to 6 weeks. The overall durations of remission in our total cases ranged from 2 to 13 months (average = 6·5) and in cases with carcinoma of the breast 7·8 months. From Table III it can be seen that these overall durations compare favourably with those reported by other workers who employed a routine of giving repeated maintenance courses at regular intervals. It is, therefore, felt that nothing is to be gained by employing a more energetic method than our " watch and palliate " policy which gives less disturbance to the patients.

SUMMARY

1. The current literature on the clinical application of 5FU has been briefly reviewed.

2. The results of the treatment of 28 Hong Kong Chinese patients suffering from various types of advanced solid cancers with 5FU alone or in combination with deep X-rays were presented. Of the two types of neoplasm, adenocarcinoma and epidermoid carcinoma, objective response was observed in 58·3 per cent of the former cases and 16·6 per cent in the latter.

3. Apart from marked anorexia, side effects were less frequently encountered among Hong Kong Chinese patients than among European patients treated elsewhere.

4. Objective response was not invariably associated with severe toxic reactions among Chinese patients. Only 3 out of 8 patients with objective response had side effects worthy of note.

5. If complete or optimum tumour regression has been achieved after a full course of 5FU, a " watch and palliate " policy which gives less disturbance to the patient is recommended in preference to the giving of repeated maintenance courses at 4–6 weeks' interval.

Our grateful thanks are due to Dr. the Hon. D. J. M. MacKenzie, the Director of Medical and Health Services of Hong Kong, for his kind permission to publish this paper, to our medical colleagues and nursing staff of the Queen Mary and Tung Wah Hospitals for their unreserved co-operation in making this clinical trial possible, to Mr. K. W. Leung, our Clinical Photographer, for preparing the photographic illustrations and, last but not least, to Miss Iris Wong for her help in typing the manuscripts.

REFERENCES

ANSFIELD, F. J., SCHROEDER, J. M. AND CURRERI, A. R.—(1962) *J. Amer. med. Ass.*, **181**, 295.

Clinical Data Sheet on 5-Fluorouracil (Ro 2-9757) by Hoffmann-LaRoche Inc. (1960)

CURRERI, A. R., ANSFIELD, F. J., McIVER, F. A., WEISMAN, H. A. AND HEIDELBERGER, C.—(1958) *Cancer Res.*, **18**, 478.

DEREN, T. L. AND WILSON, W. L.—(1960) *J. Urol.*, **83**, 390.

GOLD, G. L., HALL, T. C., SHNIDER, B. I., SELAWRY, O., COLSKY, J., OWENS, A. H. Jr., DEDERICK, M. M., HOLLAND, J. F., BRINDLEY, C. O. AND JONES, R.—(1959) *Cancer Res.*, **19**, 935.

HEIDELBERGER, C., CHAUDHURI, N. K., DANNEBERG, P., MOOREN, D., GREISBACH, L., DUSCHINSKY, R., SCHNITZER, R. J., PLEVEN, E. and SCHEINER, J.—(1957) *Nature, Lond.*, **179**, 663.

HURLEY, J. D. AND ELLISON, E. H.—(1960) *Ann. Surg.*, **152**, 568.

*Idem*, ELLISON, E. H., RIESCH, J. D. AND SCHULTE, W.—(1960) *J. Amer. med. Ass.*, **174**, 1696.

*Idem*, TRUMP, D. S., FLATLEY, T. J. AND RIESCH, J. D.—(1961) *Arch. Surg.*, **83**, 611.

KENNEDY, B. J. AND THEOLOGIDES, A.—(1961) *Ann. intern. Med.*, **55**, 719.

SCHELL, H. W. AND CRESSY, N. L.—(1962) *Amer. J. med. Sci.*, **244**, 88.

WEISS, A. J., JACKSON, L. G. AND CARABASI, R.—(1961) *Ann. intern. Med.*, **55**, 731.

WILSON, W. L.—(1960) *Cancer*, **13**, 1230.

ZUBROD, C. G.—(1961) *J. Amer. med. Ass.*, **178**, 832.